# Birch Pollen Related Pear Allergy: A Single-Blind Oral Challenge TRIAL with 2 Pear Cultivars

**DOI:** 10.3390/nu13041355

**Published:** 2021-04-18

**Authors:** Nicolette W. de Jong, Severina Terlouw, Frank E. van Boven, M.S. van Maaren, Marco W.J. Schreurs, Dianne B.P.M. van den Berg-Somhorst, Diederik Esser, Shanna Bastiaan-Net

**Affiliations:** 1 Internal Medicine, Allergology & Clinical Immunology, Erasmus MC, University Medical Centre, 3015 GD Rotterdam, The Netherlands; s.terlouw@erasmusmc.nl (S.T.); f.boven@erasmusmc.nl (F.E.v.B.); m.vanmaaren@erasmusmc.nl (M.S.v.M.); 2 Department of Immunology, Laboratory Medical Immunology, Erasmus MC, University Medical Centre, 3015 GD Rotterdam, The Netherlands; m.schreurs@erasmusmc.nl; 3 Wageningen Food & Biobased Research, Wageningen University & Research Centre, 6708 WE Wageningen, The Netherlands; dianne.somhorst@wur.nl (D.B.P.M.v.d.B.-S.); diederik.esser@wur.nl (D.E.); shanna.bastiaan@wur.nl (S.B.-N.)

**Keywords:** birch pollen, allergy, Bet v 1, OAS, pear, oral challenge

## Abstract

Approximately 70% of birch pollen allergic patients in Europe experience hypersensitivity reactions to Immunoglobulin E (IgE) cross-reactive food sources. This so-called pollen-food syndrome (PFS) is defined by allergic symptoms elicited promptly by the ingestion of fruits, nuts, or vegetables in these patients. So far, in the literature, less attention has been given to Bet v 1 cross-reactive symptoms caused by pear (*Pyrus communis*). In the Netherlands, pears are widely consumed. The primary objective of this study was to measure the type and severity of allergic symptoms during pear challenges in birch pollen allergic patients, with a positive history of pear allergy, using two different pear varieties. Fifteen patients were included, skin prick test (SPT), prick-to-prick test (PTP), specific Immunoglobulin E (sIgE), and single-blind oral challenges were performed with two pear (*Pyrus communis*) varieties: the ‘Cepuna’ (brand name Migo^®^) and the ‘Conference’ pears. All patients were sensitized to one or both pear varieties. A total of 12 out of 15 participants developed symptoms during the ‘Cepuna’ food challenge and 14/15 reacted during the ‘Conference’ challenge. Challenges with the ‘Cepuna’ pears resulted in less objective symptoms (*n* = 2) in comparison with challenges with ‘Conference’ pears (*n* = 7). Although we did not find significance between both varieties in our study, we found a high likelihood of fewer and less severe symptoms during the ‘Cepuna’ challenges. Consequently selected pear sensitized patients can try to consume small doses of the ‘Cepuna’ pear outside the birch pollen season.

## 1. Introduction

In the general population in Europe, the prevalence of birch pollen sensitization ranges from approximately 8 to 16% [1]. The prevalence of sensitization to Bet v 1, a PR-10 allergen, and the major allergen of birch pollen, is notably high among European patients with pollen allergies. In a study of 260 patients with tree pollen allergy in Germany, 92% were sensitized to Bet v 1 [2], and in a retrospective study of 854 patients with birch pollen sensitization in Italy, sensitization to Bet v 1 ranged from 53% to 95%, depending on the region [3]. Approximately 70% of birch pollen allergic patients experience hypersensitivity reactions to IgE cross-reactive food sources [4]. This so-called pollen-food syndrome (PFS) is defined by allergic symptoms elicited promptly by the ingestion of fruits, nuts, or vegetables in patients with seasonal allergic rhinoconjunctivitis (SAR) [5]. Patients are sensitized to pollen allergenic molecules highly cross-reacting with their homologs in the offending foods [6,7]. Symptoms of PFS are often restricted and isolated to the oral cavity and include labial and oropharyngeal pruritus, paraesthesia, and angioedema of the oral mucosa, tongue, lips, palate, and oropharynx, or laryngeal tightness, which altogether are labeled as oral allergy syndrome (OAS). Gastrointestinal symptoms and, rarely, life-threatening wheezing and anaphylaxis, occur in less than 10% of patients [8]. Bet v 1, a PR-10 type of protein, is the most prevalent cause of cross-sensitization. The PR-10 related reactions are mainly in response to *Rosaceae* fruits (i.e., apples) and nuts (i.e., hazelnut). The most frequently described cross-reacting fruits are apple, peach, cherry, and apricot, but a wide range of fruits contain Bet v 1 homologs. So far, in the literature, less attention has been given to Bet v 1 cross-reactive symptoms caused by pear (*Pyrus communis*). Most larger prevalence studies in Europe did not include pear while in most apple allergic patients pear also causes symptoms [9]. One systematic review by Zuidmeer et al. [10] found a study on pear allergy [11] reporting 0.3% pear allergy in Germany. Furthermore, Rodriquez et al. [12] performed skin prick tests in 26 patients in Spain with adverse reactions to *Rosaceae* fruits, and 18 patients with positive SPT for apple appeared to be positive for pear as well (69%). In the Netherlands, pears are widely consumed. Each household consumes an average of 4.7 kilos of pear per year and pear is in a solid third place in the Dutch fresh fruit top 10 [13]. In the Erasmus MC Rotterdam, SPT with pear is regularly positive in birch pollen allergic patients but clinical relevance is often unclear. Diagnosis of pear allergy has to be confirmed by a double-blind placebo-controlled food challenge. The primary objective of this study was to measure type and severity of allergic symptoms during pear challenges in birch pollen allergic patients, with a positive history of pear allergy, using two different pear varieties.

## 2. Materials and Methods

### 2.1. Patients

Adult patients visiting the outpatient clinic of the department of Allergology of the Erasmus MC with a doctor’s diagnosed birch pollen allergy and a positive history of pear allergy were asked to participate in the study. The patients were approached from August 2019 and inclusion started on 1 October 2019 till 1 February 2020. Medical ethical approval was received in August 2019; registered as METC NL70165.078.09. The purpose was to perform the study just outside the ‘birch pollen season’ (February to May) to circumvent that participating patients could not stop their anti-histamines, and/or preventing possible bias in patients having more symptoms during that season.

### 2.2. Pears

Two pear (*Pyrus communis*) varieties ‘Cepuna’ (brand name Migo^®^) and ‘Conference’ pear were tested according to a normal consumer simulation consisting of simulating refrigerated transport to the supermarket and consumer storage in a fruit bowl. Pears were acclimatized in advance (via ‘chambreren’) by GKE NV and delivered ready for consumption every week by courier. Pears delivered were stored in the refrigerator. The evening before inclusion, the number of pears needed was brought to room temperature. Before use, the pears were rinsed under the tap with water as in a home situation.

### 2.3. Skin Test

Skin prick test (SPT) and prick-to-prick (PTP) tests were performed with both pear varieties on the forearm during the first day of the oral challenge, with pear juice and fresh pears respectively, next to 2 positive controls (histamine), birch pollen extract, and a negative control (ALK-Abello; Almere, the Netherlands). The difference between SPT and PTP is that in the PTP test, the needle is first pricked into the fresh intact unpeeled pear near the stalk [14] and the juice sticking to the needle is subsequently transferred into the skin of the participant. The SPT was performed by applying a drop of whole fresh pear juice on the skin of the volar aspect of the forearm. Twenty minutes after the skin tests, the contours of the wheal were encircled with a fine-tip pen and transferred to a record sheet by translucent tape [15]. Subsequently, the surface was measured with an area scanner and compared with the positive control which gives the HEP index score as described by van der Valk et al. [15]. No threshold values have yet been defined for the SPT and PTP HEP index values for pear allergy. SPT and PTP tests were considered positive when ≥3 mmØ [16,17].

### 2.4. Specific Serum IgE

Specific Immunoglobulin E (sIgE) antibody concentration was measured in blood serum. Specific serum IgE for pear allergen extract (f94), birch pollen allergen component Bet v 1 PR10 (t215), peach allergen component Pru p 3 LTP (f420), and grass pollen allergen component Phl p 12 profilin (g212) were measured by fluorescence immunoassay (FEIA) using the ImmunoCAP™ test system, according to the manufacturer’s instructions (Thermo Fisher Scientific, Upsala, Sweden). sIgE concentration was considered positive when >0.35 KU/L.

### 2.5. Single Blind Oral Challenges

As validated recipes for double-blind placebo-controlled food challenges with pear do not exist, we performed single-blind oral pear challenges. The oral pear challenges consisted of two-day admissions in the outpatient clinic, in which the pear ‘Cepuna’ was tested on one day and the pear ‘Conference’ on the other day. For each participant, the order in which each pear variety was tested was randomized. The single-blind oral food challenge consisted of five doses of pear, including the peel as described by Rodriguez et al. [12]. The doses were calculated using the ‘voedingswaardetabel.nl’ site. According to this site, pears, in general, contain 0.5 g protein/100 g pear. Following the PRACTALL guidelines, we challenged 10 µg, 30 µg, 100 µg, 300 µg, and 1000 µg protein, which resulted in the following dose series: 2, 6, 20, 60, and 200 g of pear. The person that weighed the pear doses and prepared the challenge was not the same person as the one who gave the pear pieces to the patient. So, the nurse was not blinded but was ignorant about which variety was given to the patient. The symptoms during the challenge were recorded according to the PRACTALL guidelines, [18] and scored as mild, moderate, or severe. The challenge was stopped as soon as the participant responded three consecutive times with subjective symptoms to a certain dose and was stopped immediately in case the patient reacted with objective symptoms, as based on the reference of Sampson et al. [18]. This means that not every patient consumed the last doses, dose four and dose five. To minimize sensory perception between the pear varieties, patients were blindfolded and wore a nose clip during the oral challenge (single-blind). After the challenge, participants remained in the clinic for 2 h to monitor possible reactions. Twenty-four hours after the challenge the patients were contacted by telephone to register possible late reactions. Subjective symptoms were recorded as itching in the mouth and on the lips, in the ears, nose, or eyes, and nausea. Objective symptoms are seen as the more serious symptoms and consist of itchy skin or red skin (urticaria), wheezing, and laryngeal symptoms.

### 2.6. IgE-Immunoblotting/SDS Page/Electrophorese

Pears were cut into an upper and a lower part. The upper quarter of the pear, without the inner core, was cut into pieces and snap-frozen in liquid nitrogen. From the bottom part, only the peeled skin (still containing a little bit of flesh) was collected and combined with the upper quarter of the pear to obtain an equal amount of flesh versus peel material. Per variety, 10 pears were sampled to obtain a representative sample batch. Subsequently, the sampled material was ground under liquid nitrogen using an IKA mill and the acquired powder was stored at −80 °C until further use. Total protein was extracted using the method described by Vieths et al. [19] with slight adjustments. Since pears are high in polyphenol content, and these can interfere with protein isolation [20], ground pear samples were homogenized in acetone/dry ice and incubated overnight while stirring and cooled by dry ice. Precipitates were washed twice with acetone/dry ice, and once with acetone/diethyl ether/dry ice (1:1, *v*/*v*, −60 °C). Subsequently, precipitates were filtered (Whatman^®^; 595½, ø240 mm, Pfullingen, Germany), lyophilized, and stored at −20 °C until protein extraction. Total protein extracts were obtained by extraction with 0.001 M potassium phosphate (mix K2HPO4 and KH2PO4) buffer pH7.4, containing 0.15 M NaCl by overnight incubation at 4 °C on a stirring weal (2 g of acetone powder/30 mL extraction buffer). The next day, samples were centrifuged (4 °C, 60 min, 4700 rpm) and the protein supernatants were concentrated using 3 kDa Amicon concentrators (Merck Millipore; Tullagreen, Ireland). The protein concentration was determined using Bradford assay (Thermo Fisher Scientific Inc., Madison, WI, USA) according to the manufacturer’s instructions using bovine serum albumin (BSA) as a protein standard. IgE-Immunoblotting was performed as described previously [21]. In brief, 20 µg of pear protein concentrate was separated by SDS PAGE on Bolt^TM^ 4–12% Bis-Tris Plus gels next to a Precision Plus Protein Dual Xtra Standard molecular weight marker (Bio-rad, Hercules, CA, USA) according to the manufacturer’s instructions (Invitrogen, Carlsbad, CA, USA) and either stained by Simply Blue safe stain (Thermo Fisher Scientific Inc.) or transferred to a 0.2 μm nitrocellulose membrane (LKB, Bromma, Sweden) by Tris-glycine buffer (25 mM Tris, 190 mM glycine, 0.1% SDS, 20% methanol) for 36 min at 70 V using a Criterion blotter (Bio-rad). The transfer was verified using the MemCode Reversible Protein Stain Kit (Thermo Fisher Scientific Inc.). After blocking in 3% BSA, blots were incubated overnight with 1:5 diluted patient serum (2 mL). The first and secondary antibodies used were the polyclonal rabbit anti-IgE antibody from Dako (1:1000; Glostrup, Denmark) and AP-conjugated polyclonal goat anti-rabbit antibody from Sigma Aldrich (1:20,000; Saint Louis, MO, USA). Blots were stained for 30 min in 20 mL NBT/BCIP staining solution (Sigma Aldrich; St. Louise, MO, USA). Imaging and analysis of antibody binding were performed using a Universal Hood III and Image Lab 4.1 software (Bio-Rad; Hercules, CA, USA).

### 2.7. Statistics

One of the objectives was to compare both pear varieties based on allergenicity. Calculating Bayes factors compared the differences in the pear outcomes. The Bayes factor (BF) is a likelihood ratio of a null hypothesis and an alternative [22]. Evidence for the alternative hypothesis (H1) was set as BF > 3 (moderately), BF > 10 (strongly), BF > 30 (very strong), and BF > 100 (extremely), and evidence for the null hypothesis (H0) was set as BF < 1/3. Dependencies between pear outcomes were analyzed by the Fisher exact test. The classification concerns included the difference in the amount of pear consumed and the various scores. All calculations were performed with R, in the Fisher exact test, *p* < 0.05 is considered to be statistically significant.

## 3. Results

### 3.1. Patients

Based on the medical history of patients registered at Erasmus MC, a total of 74 patients with birch pollen allergy were approached, of which 17 were included (20%). Thirty-one patients did not want to participate despite previous symptoms while eating pear, while 28 patients had tree pollen allergies without symptoms when consuming pear or other fruit. Of the 17 included patients, two dropped out: one patient was negative in SPT and PTP on both pear varieties tested, and one patient did not attend the second visit. Finally, fifteen patients were included in the study, 80% of which were female. The average age was 37 years (range 20–64 years). Eleven patients did not consume pear. Four patients indicated eating, very occasionally, processed pears (heated, cooked). Of the eleven patients who did not eat pears, 10 patients had eliminated pears from their diet for >3 years, and one patient less than 3, but more than 2 years from their diet (Table 1).

### 3.2. SPT/PTP

The SPT HEP index for ‘Cepuna’ pear and ‘Conference’ pear was on average 0.20 (range 0–0.58) and 0.22 (range 0–0.83), respectively. The prick-to-prick (PTP) test HEP index with ’Cepuna’ and ‘Conference’ pear averaged 0.81 (range 0–2.57) and 0.61 (range 0.13–2.37), respectively. SPT was negative (<3 mm Ø) in 9 cases (60%) for both pear varieties. PTP was negative with ‘Cepuna’ in 2 cases (13%) and with ‘Conference’ in 1 case (6%). All patients were sensitized to pear in at least one of the tests (SPT, PTP, or sIgE). SPT with birch pollen was positive in 14/15 patients with an average HEP of 1.28 (Table 2). A test of difference produced a BF of 29 (BF > 10 = strongly) in favor of a PTP being larger than SPT in ‘Cepuna’ as well as ‘Conference’. The mean of SPT and PTP did not differ between ‘Cepuna’ and ‘Conference’ (BF = 1.35). SPT and PTP were not associated with the challenge outcomes for ‘Cepuna’ as well as ‘Conference’ pears (*p* = 0.13 to 1.0 resp.).

### 3.3. sIgE

One patient refused to give blood (nr 9), so 14 sera for sIgE measurements were available (Table 2). The sIgE serum concentration for pear was on average 2.91 KU/L (range 0–16.7 KU/L) and was negative (<0.35 KU/L) in four cases (29%). These patients may be solely sensitized to Pyr c 1, and it is not clear whether it is in the f94 pear extract. For Bet v 1 (PR10) sIgE, the average was 24.86 KU/L (range 0–102.5 KU/L) and negative in one case (7%). Furthermore, sIgE to Pru p 3 (LTP) was positive in 2 patients, nr 1 and 10 with values of 2.1 KU/L and 91.6 KU/L respectively. SIgE to Phl p 12 (profilin) was also positive in 3 cases: nrs 5, 13 and 15: 4.8; 0,7 and 27.2 KU/L respectively. SIgE serum concentration was not associated with SPT, PTP, and challenges for both the ‘Cepuna’ and ‘Conference’ pear (*p* = 0.15 to 1.0).

### 3.4. Pear Challenge

Twelve out of fifteen participants (80%) developed symptoms during the ‘Cepuna’ food challenge. Three participants could eat the whole ‘Cepuna’ pear without symptoms (nrs 5, 6, and 12). Fourteen out of fifteen participants (93%) developed symptoms during the ‘Conference’ food challenge, in which only one participant (nr 9) could eat the whole pear without symptoms. None of the patients showed a late reaction (24 h after the food challenge) after either challenge. The BF of a reduced number of positive challenges was 8 for ‘Cepuna’ pear, and 0,4 for ‘Conference’ pear (Table 2).

Challenges with the ‘Cepuna’ pears resulted in less objective symptoms (two patients) in comparison with challenges with ‘Conference’ pears (seven patients) (BF = 4192). Most of the scores were assessed as mild (score 1). During the ‘Cepuna’ challenge, four patients scored moderate (score 2) for itchy mouth (nrs 3, 8, 9, and 14) and one patient scored moderate (score 2) for wheeze and larynx symptoms (nr 4). During the ‘Conference’ challenge, three patients scored moderate (score 2) for itchy mouth (nrs 7, 12, and 15) and one patient for nose and/or ears symptoms (nr 7). One patient scored severe (score 3) for itchy mouth (blisters) (nr 6) (Table 3).

Overall, four patients were treated with antihistamine for their allergic reaction (nrs 1, 2, 3, and 4) because they asked for it. The wheeze and larynx symptoms were mild and consequently, no adrenaline or corticosteroids were administered.

### 3.5. Immunoblotting

For Western blot analysis, 15 µg of total protein concentrates were separated on SDS PAGE under reducing conditions and transferred to membranes that were subsequently incubated with the serum of each of the participating patients (except for pt nr 9).

**Figure 1:**
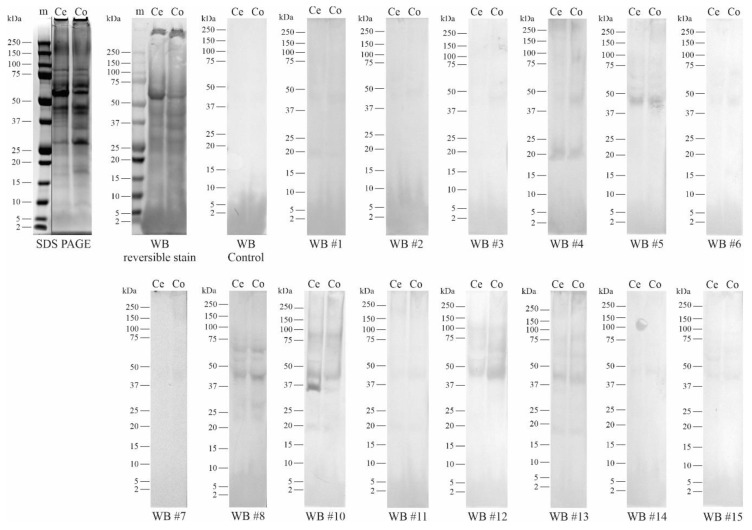
Western blots (WB) of ‘Cepuna’ and ‘Conference’ pear total protein concentrate using the patient serum. The WB control blot was exposed to buffer instead of serum. Patient numbering is indicated by #.

The blots incubated with patient sera indicate that most pear allergic individuals carry IgE antibodies against relatively moderate molecular weight (MW) proteins (between 25 and 75 kDa) while a few patients also react to proteins in the small MW range of 15–25 kDa (Figure 1 and Supplementary Table S1).

All patients, except patient nr 15, seem to have IgE antibodies that bind (in more or less intensity) a protein band with an estimated MW of ~45 kDa. The majority of patient sera bound to protein bands with an estimated MW of ~19 kDa and ~55 kDa (Table S1 Supplementary).

Patients nrs 5, 6, and 12 did not show an allergic response to the consumption of ‘Cepuna’ pear while responding to ‘Conference’ pear upon the first or second dose. However, in Western blot, these patient sera recognized the ~45 kDa band with almost equal intensity for both pear varieties, suggesting that IgE binding to this protein band might be clinically irrelevant.

Up till today, four allergens are officially identified in pear: Pyr c 1.0101, Pyr c 3.0101, Pyr c 4.0101, and Pyr c 5.0101, which represent the protein allergen types Bet v 1-like, non-specific lipid transfer protein (nsLTP), profilin and isoflavone reductase, respectively (Table 4). In apple, another protein allergen type is known, named thaumatin, which might also be present in pear but has not yet been identified.

The estimated ~35 kDa band, recognized by patient sera nr 10 and nr 14, might be Pyr c 5.0101 or another isoflavone reductase isoform, based on the similarity of MW. The Bet v-like allergen in pear, Pyr c 1.0101, might represent the ~19 kDa band on gel, while profilin (Pyr c 4.0101) might represent the estimated ~15 kDa band, slightly bound by patient serum nr 6. The birch pollen sIgE measurements back up the hypothesis that the ~19 kDa band might represent Pyr c 1.0101, as patient nr 4, who showed the highest amount of birch pollen sIgE (102.5 kU/L), also showed the highest band intensity on the blot. Serum IgE from patient nr 8 seems to bind a ~2 kDa protein band, which might represent a thaumatin type of protein, as identified as an allergen in apple (Mal d 2.0101). The identity of the high MW bands bound by the patient sera IgE is impossible to estimate but could be identified in the future by performing an LC-MS/MS analysis.

## 4. Discussion

In this study, we describe a group of birch pollen allergic patients, suffering from oral allergy symptoms during the consumption of pear. This Bet v 1 (PR-10) related fruit allergy is hardly described. According to Beyer et al. [23], the foods that most frequently elicit allergic reactions in birch pollen allergic patients were apple (78%), carrot (52%), and peach (49%). However, in the same study, pear comes close to these numbers at 36%. This study in Germany focused on food allergy-related quality of life (FAQL) in birch pollen-associated food allergy (FA) symptoms. The mean food allergy Quality of Life Questionnaire—Adult form (FAQLQ-AF) score was 3.7. This shows the extent of everyday impairment in this group of patients with food allergies.

Pear is becoming popular in Europe and consumed widely because of its nutritional benefits [24]. We included patients who previously reported symptoms after consumption of pear and in most cases, this could be confirmed in the single-blind oral challenges. Four patients had a negative challenge to one of both varieties, of which two patients appeared to have low to negative sensitization profiles. At the same time, all patients reacted to one of both pears, irrespective of their sensitization pattern. Remarkably, most patients experienced subjective symptoms directly after consuming dose one (10 µg pear). Patients with OAS recognized these symptoms; however, challenges were continued with the next doses, as subjective symptoms should occur on three consecutive doses to be positive, following the PRACTAL guidelines. Nevertheless, v. Erp et al. showed that subjective symptoms are significantly associated with disagreement when assessed by different clinical experts [25]. So, variability in the interpretation of food challenge outcomes exists, especially when objective symptoms are absent.

Overall, in this study, besides subjective symptoms, objective symptoms also occurred in seven cases, but we cannot neglect that subjective symptoms are prone to interpretation bias by the patient as well as the researcher. Furthermore, a shortcoming in this study might be the open challenges. As earlier described, up to 12.9% of placebo reactions can appear during food challenges and this is usually captured by performing double-blind placebo-controlled food challenges (DBPCFC) [26]. Unfortunately, double-blind challenges with pear were not possible, as validated recipes with e.g., the right matrix do not exist. To capture this problem the patients were blindfolded and used a nose clip, and the nurse who provided the pear doses was not the same as the nurse who assessed the symptoms. Nevertheless, we are aware the study is most likely somewhat biased by this phenomenon and that taste preference could have played a role in the assessment of the symptom scores.

To statistically compare the several types of data we used the Bayes factor (BF), which is a weighted average likelihood factor of a particular hypothesis. The BF of a reduced number of positive challenges was 8 for ‘Cepuna’ pear, and 0.4 for ‘Conference’ pear and challenges with the ‘Cepuna’ pears resulted in less objective symptoms in comparison with challenges with ‘Conference’ pears (BF = 4192 (extremely strong)). In both cases, the data are in favor of the ‘Cepuna’ pear in comparison to the ‘Conference’ pear. Particularly, the likelihood of a difference is extreme in the case of objective symptoms.

SPT was negative in nine cases with both pear varieties, while PTP was only negative in two cases. In two of those negative SPTs, a negative challenge confirmed the negative sensitization, but the other seven cases were positive in the oral challenge. A test of difference produced a BF of 29 in favor of a PTP being larger than SPT in ‘Cepuna’ as well as ‘Conference’. So far, these comparisons of skin tests with pear have never been made. PTP is widely accepted as a reliable tool for measuring sensitization to fruits in patients with OAS [27]. Our results also tie in well with the study of Vlieg-Boerstra et al. who concluded that SPT was not useful to assess the allergenicity of 68 apple cultivars [28]. So, in this study, we again confirmed that PTP with fresh fruit is the best method to be used in the diagnosis of fruit food allergy. The study was performed in birch pollen allergic patients and cross-sensitization to PR-10 allergens are most likely. Symptoms caused by these PR-10 allergens are often subjective and mild (OAS) [29]. Unfortunately, we could only slightly confirm the presence of these PR10 antibodies in the sera of the patients in immunoblot, as binding at 17.5–19 kDa was only evident in a few patients. This might be caused by the extraction method of the pears, which was not described in earlier literature, and apple protocols were used as an alternative [19], which might have resulted in an underrepresentation of Pyr c 1 in our extract [18]. Such is also often the case in commercial diagnostic fruit extracts including f94, which might also explain why four patients were negative for the pear f94 sIgE measurements while being positive for birch pollen sIgE (Table 2). Another explanation could be that most of these patients recognize PR-10 conformational epitopes, which are (partly) destroyed under reducing SDS PAGE conditions. In follow-up studies, native or non-reducing conditions might be considered to study Pyr c 1 IgE binding in more detail. Furthermore, it might be possible that the patients are sensitized to proteins in the pear with a higher molecular weight, e.g., Pyr c 5.0101 as in some cases, binding in immunoblot is present to high molecular weight proteins. We did not quantify sIgE to this 33.8 kDa protein. However, as these patients are all birch pollen allergic, their pear allergy is most likely caused by cross-reactivity to PR10 allergens [4]. Although sensitized to PR-10 allergens, several patients in this study also reacted with objective symptoms, which are usually seen as more severe. This is quite remarkable. These objective symptoms are often caused by non-PR-10 allergens e.g., nsLTPs. In our study, only one patient (nr 10) was sensitized to nsLTP (Pru p 3) allergens with a high sIgE value of 91.6 KU/L. This patient reacted on dose 1 with subjective as well as objective symptoms (laryngeal symptoms) to both pear varieties. In contrast, in immunoblot, we could not detect IgE binding at an MW band migrating at around 11.4 kDa (nsLTP). Le et al. [30] found comparable results in a Dutch population suffering from birch pollen-related apple allergy. Of the 14 patients, only one was positive for nsLTP (Pru p 3) sIgE. Although anaphylaxis did not occur in our study with pear challenges, especially for the ‘Conference’ pear we found seven patients with skin symptoms, wheeze, and laryngeal symptoms. The wheeze and larynx symptoms were mild and consequently, no adrenaline or corticosteroids were administered; nevertheless, PR-10 pear proteins appear to be able to cause these objective symptoms after pear consumption [31]. On Western blot, most patient sera bound ‘Cepuna’ and ‘Conference’ protein bands in equal intensity, making it difficult to correlate these IgE binding results to the single-blind oral challenge results or the PTP, SPT, or sIgE measurements. Although the protein content in both pear varieties differed only slightly (0.315% ± 0.007% and 0.330% ± 0.006% for ‘Cepuna’ and ‘Conference’, respectively (DUMAS, Nf6.25); *p* < 0.05), the protein yield differed by a factor of 1.6 (2.8 mg versus 4.5 mg respectively), which might indicate that proteins in ‘Cepuna’ are more difficult to extract or less bio-available. This difference in protein availability might perhaps also occur when pears are eaten, but proving this would require further research. Differences in allergenicity may also be influenced by matrix components other than allergen content, e.g., the polyphenol content [32], which was not tested for in this study. We are aware that the applied enzyme-based immunoblot detection technique limits the sensitivity of the overall detection signal which could have restricted our data analysis especially in the case of low KU/L sIgE titers. To circumvent these limitations, future studies could consider X-ray film using radio-labeled antibodies to increase detection sensitivity. In addition, gradient gels specifically for low MW protein separation can be considered to increase sensitivity in the 7–35 kDa range, given that the known pear allergens Pyr c 1 to 5 run in this range (Table 4). We compared two pear varieties. This study was industry initiated and their hypothesis was that the ’Cepuna’ pear was less allergenic. They regularly received signals from allergic patients in the Netherlands who experienced less to no symptoms during consumption of this pear. There was a very strong likelihood of fewer symptoms during ‘Cepuna’ pear challenges and even negative in three cases. In addition, the ‘Cepuna’ pear caused objective symptoms in only two patients, versus seven patients during the ‘Conference’ challenge. The burden of pollen-related food allergy is often underestimated in patients with a multi-fruit allergy, and therefore it is of the highest interest to find one fruit that can be consumed. Fruits contain all kinds of nutrients and vitamins that are indispensable in the daily diet. Food allergic patients are interested in having low-allergen food available and want to eat the food they are allergic to [33]. Kootstra et al. [34] compared different apple cultivars and found that more than half (53%) of the patients (*n* = 15) could consume the ‘Santana’ apple without symptoms (*p* = 0.02) [34].

## 5. Conclusions

Although we did not find a significant difference in symptoms during single-blind oral challenges between both pear varieties in our study, we found a very high likelihood of fewer symptoms during the ‘Cepuna’ challenges. Consequently selected patients can try to consume small doses of the ‘Cepuna’ pear outside the birch pollen season.

## Figures and Tables

**Table 1:** Characteristics of the patients.

		n	%
Numbers included		15	
Patients	Female	12	80%
	Mean age/range	37	18–65
Inhalant allergy	HDM	11	73%
	GP	11	73%
	BP	15	100%
Food allergy	>1 allergen	1	7%
	>2 allergens	1	7%
	>3 allergens	13	87%
Current Pear consumption	No	11	73%
	Yes (processed)	4	27%
History of Symptoms	Itchy mouth	14	93%
	Skin	0	0%
	Wheeze	3	20%
Anti-allergic medication use		12	80%

HDM: house dust mite; BP: birch pollen; GP: grass pollen.

**Table 2:** Results of the SPT, PTP, sIgE, and open single-blind challenges.

	SPT	PTP	SPT	PTP	SPT	sIgE	sIgE	Single Blind Oral Challenge			
	Cepuna		Conference		Bp	Pear	Bp	Cepuna		Conference	
nrs	HEP	HEP	HEP	HEP	KU/L	KU/L	KU/L	pos/neg	dose*	pos/neg	dose*
1	neg	0.90	neg	0.25	0.61	0.6	19.2	pos	3	pos	1
2	neg	0.40	neg	0.28	1.32	2.1	12.4	pos	1	pos	1
3	0.58	2.57	0.83	1.17	5.06	0.4	16.2	pos	1	pos	5
4	0.50	1.21	neg	2.37	2.42	16.7	102.5	pos	1	pos	1
5	neg	0.51	neg	0.88	0.16	0.8	29.6	neg	neg	pos	1
6	neg	0.30	neg	0.38	1.02	neg	11.3	neg	neg	pos	2
7	0.25	1.09	0.46	0.77	1.52	0.7	15.6	pos	1	pos	2
8	neg	0.71	neg	0.24	neg	neg	8.8	pos	4	pos	3
9	neg	neg	0.14	0.34	0.61	NA	NA	pos	2	neg	neg
10	neg	1.33	neg	0.14	0.62	8.6	3	pos	1	pos	1
11	neg	neg	neg	neg	1.43	5.1	46.3	pos	1	pos	1
12	0.38	1.32	0.37	0.89	1.18	neg	51.5	neg	neg	pos	1
13	neg	0.32	neg	0.35	0.71	2.1	25	pos	5	pos	2
14	0.22	0.38	0.21	0.37	1.20	neg	6.7	pos	1	pos	1
15	0.24	0.53	0.20	0.69	0.94	0.6	neg	pos	5	pos	1

SPT: skin prick test; PTP: prick-to-prick test; sIgE: serum immune globulin E; HEP: histamine equivalent prick index; pos: positive; Neg: < 3 mmØ; dose*: lowest dose during the pear challenge that the patient reacted to; NA: not available. Bp: birch pollen.

**Table 3:** Symptoms during pear challenges.

	‘Cepuna’		‘Conference’	
PT nr	Subjective	Objective	Subjective	Objective
1	IM		IM	SK, LA
2	IM, IE		IM, IE	LA
3	IM/2, IN		IM	
4	IM	WH/2, LA/2	IM	
5	-------		IM	
6	-------		IM/3	
7	IM, IE, IN		IE, IN/2	WH, LA
8	IM/2		IM	
9	IM/2		--------	
10	IM	LA	IM	LA
11	IM, IN		IM, IN	SK, LA
12	--------		IM/2, IN	WH
13	IM		IM, N	
14	IM/2, IN, N		IM, IN, N	LA
15	IM		IM/2	

Score: All patients score mild/1, except when given/2 for moderate, or/3 for severe. IM: itchy mouth; IE: itchy ears; IN: itchy nose and ears; N: nausea; SK: skin; WH: wheeze; LA: laryngeal.

**Table 4:** Pear (Pyrc) and apple (Mald) allergens described in the Allergome database (http://www.allergome.org/index.php, accessed on 10 October 2020).

Allergen Name	Protein Type	kDa Based on AA Sequence	kDa without Signal Peptide
Pyr c 1.0101	Bet v 1-like	17,581	
Pyr c 3.0101	nsLTP	11,463	9125
Pyr c 4.0101	Profilin	14,064	
Pyr c 5.0101	Isoflavone reductase	33,823	
Mal d 2.0101	Thaumatin	25.7	23,211

## Data Availability

The original database ie not available online.

## Supplementary Materials

The following are available online at https://www.mdpi.com/article/10.3390/nu13041355/s1. Table S1: Indicative molecular weight of protein bands bound by IgE antibodies in patient sera; protein bands corresponding to the Western blots in Figure 1. Ce: ‘Cepuna’ pear; Co: ‘Conference’ pear; WB: Western blot; the number of x’s indicate the (by eye) estimated intensity of the coloration. Serum nr 9 was not available for Western blotting.

## References

[1] Biedermann T., Winther L., Till S.J., Panzner P., Knulst A., Valovirta E. (2019). Birch pollen allergy in Europe. Allergy.

[2] Canis M., Gröger M., Becker S., Klemens C., Kramer M.F. (2011). Recombinant marker allergens in diagnosis of patients with allergic rhinoconjunctivitis to tree and grass pollens. Am. J. Rhinol. Allergy.

[3] Ciprandi G., Comite P., Mussap M., De Amici M., Quaglini S., Barocci F., Marseglia G.L., Scala E. (2016). Profiles of Birch Sensitization (Bet v 1, Bet v 2, and Bet v 4) and Oral Allergy Syndrome Across Italy. J Investig. Allergol. Clin. Immunol..

[4] Geroldinger-Simic M., Zelniker T., Aberer W., Ebner C., Egger C., Greiderer A., Prem N., Lidholm J., Ballmer-Weber B.K., Vieths S. (2011). Birch pollen-related food allergy: Clinical aspects and the role of allergen-specific IgE and IgG4 antibodies. J. Allergy Clin. Immunol..

[5] Katelaris C.H. (2010). Food allergy and oral allergy or pollen-food syndrome. Curr. Opin. Allergy Clin. Immunol..

[6] Valenta R., Niederberger V. (2007). Recombinant allergens for immunotherapy. J. Allergy Clin. Immunol..

[7] Werfel T., Asero R., Ballmer-Weber B.K., Beyer K., Enrique E., Knulst A.C., Mari A., Muraro A., Ollert M., Poulsen L.K. (2015). Position paper of the EAACI: Food allergy due to immunological cross-reactions with common inhalant allergens. Allergy.

[8] Mari A., Ballmer-Weber B.K., Vieths S. (2005). The oral allergy syndrome: Improved diagnostic and treatment methods. Curr. Opin. Allergy Clin. Immunol..

[9] Burney P.G., Potts J., Kummeling I., Mills E.N., Clausen M., Dubakiene R., Barreales L., Fernandez-Perez C., Fernandez-Rivas M., Le T.M. (2014). The prevalence and distribution of food sensitization in European adults. Allergy.

[10] Zuidmeer L., Goldhahn K., Rona R.J., Gislason D., Madsen C., Summers C., Sodergren E., Dahlstrom J., Lindner T., Sigurdardottir S.T. (2008). The prevalence of plant food allergies: A systematic review. J. Allergy Clin. Immunol..

[11] Zuberbier T., Edenharter G., Worm M., Ehlers I., Reimann S., Hantke T., Roehr C.C., Bergmann K.E., Niggemann B. (2004). Prevalence of adverse reactions to food in Germany-a population study. Allergy.

[12] Rodriguez J., Crespo J.F., Lopez-Rubio A., De La Cruz-Bertolo J., Ferrando-Vivas P., Vives R., Daroca P. (2000). Clinical cross-reactivity among foods of the Rosaceae family. J. Allergy Clin. Immunol..

[13] (2018). Meest Geconsumeerde Fruitsoorten. Resultaten van VCP 2012–2014. Rijksinst. voor Volksgezond..

[14] Vlieg-Boerstra B.J., van de Weg W.E., van der Heide S., Dubois A.E. (2013). Where to prick the apple for skin testing?. Allergy.

[15] van der Valk J.P., Gerth van Wijk R., Hoorn E., Groenendijk L., Groenendijk I.M., de Jong N.W. (2015). Measurement and interpretation of skin prick test results. Clin. Transl. Allergy.

[16] Dreborg S. (1993). Allergen standardisation and skin test: EAACI position paper. Allergy.

[17] Dreborg S. (1993). Skin testing. The safety of skin tests and the information obtained from using different methods and concentrations of allergen. Allergy.

[18] Sampson H.A., Gerth van Wijk R., Bindslev-Jensen C., Sicherer S., Teuber S.S., Burks A.W., Dubois A.E., Beyer K., Eigenmann P.A., Spergel J.M. (2012). Standardizing double-blind, placebo-controlled oral food challenges: American Academy of Allergy, Asthma & Immunology-European Academy of Allergy and Clinical Immunology PRACTALL consensus report. J. Allergy Clin. Immunol..

[19] Vieths S., Schöning B., Petersen A. (1994). Characterization of the 18-kDa apple allergen by two-dimensional immunoblotting and microsequencing. Int. Arch. Allergy Immunol..

[20] Suleria H.A.R., Barrow C.J., Dunshea F.R. (2020). Screening and Characterization of Phenolic Compounds and Their Antioxidant Capacity in Different Fruit Peels. Foods.

[21] Bastiaan-Net S., Reitsma M., Cordewener J.H.G., van der Valk J.P.M., America T., Dubois A.E.J., Gerth van Wijk R., Savelkoul H.F.J., de Jong N.W., Wichers H.J. (2019). IgE Cross-Reactivity of Cashew Nut Allergens. Int. Arch. Allergy Immunol..

[22] Makowski D., Ben-Shachar M.S., Chen S.H.A., Lüdecke D. (2019). Indices of Effect Existence and Significance in the Bayesian Framework. Front. Psychol..

[23] Beyer S., Franke A., Simon J.C., Treudler R. (2016). Measurement of health-related quality of life in adult patients with birch pollen-associated food allergy. J. Dtsch. Dermatol. Ges..

[24] Reiland H., Slavin J. (2015). Systematic Review of Pears and Health. Nutr. Today.

[25] van Erp F.C., Knulst A.C., Meijer Y., Gabriele C., van der Ent C.K. (2014). Standardized food challenges are subject to variability in interpretation of clinical symptoms. Clin. Transl. Allergy.

[26] Vlieg-Boerstra B.J., van der Heide S., Bijleveld C.M., Kukler J., Duiverman E.J., Dubois A.E. (2007). Placebo reactions in double-blind, placebo-controlled food challenges in children. Allergy.

[27] Vlieg-Boerstra B.J., van de Weg W.E., van der Heide S., Skypala I., Bures P., Ballmer-Weber B.K., Hoffmann-Sommergruber K., Zauli D., Ricci G., Dubois A.E. (2013). Additional indications for the low allergenic properties of the apple cultivars Santana and Elise. Plant Foods Hum. Nutr..

[28] Vlieg-Boerstra B.J., van de Weg W.E., van der Heide S., Kerkhof M., Arens P., Heijerman-Peppelman G., Dubois A.E. (2011). Identification of low allergenic apple cultivars using skin prick tests and oral food challenges. Allergy.

[29] Turner P.J., Dawson T.C., Skypala I.J., Fox A.T. (2015). Management of pollen food and oral allergy syndrome by health care professionals in the United Kingdom. Ann. Allergy Asthma Immunol..

[30] Le T.M., van Hoffen E., Lebens A.F., Bruijnzeel-Koomen C.A., Knulst A.C. (2013). Anaphylactic versus mild reactions to hazelnut and apple in a birch-endemic area: Different sensitization profiles?. Int. Arch. Allergy Immunol..

[31] Skypala I.J. (2020). Can patients with oral allergy syndrome be at risk of anaphylaxis?. Curr. Opin. Allergy Clin. Immunol.

[32] Russell W.R. (2009). Phenolic acid content of fruits commonly consumed and locally produced in Scotland. Food Chem..

[33] Miles S. (2004). Attitudes towards low-allergen food in food allergic consumers. Nutr. Food Sci..

[34] Kootstra H.S., Vlieg-Boerstra B.J., Dubois A.E. (2007). Assessment of the reduced allergenic properties of the Santana apple. Ann. Allergy Asthma Immunol..

