# Supplementary material for: Birch Pollen Related Pear Allergy: A Single-Blind Oral Challenge TRIAL with 2 Pear Cultivars
Source: Nutrients. 2021 Apr 18;13(4):1355. doi: 10.3390/nu13041355 (PMC8073155; doi:10.3390/nu13041355)
Supplement: Supplementary file 1 [file nutrients-13-01355-s001.zip › nutrients-1142898-supplementary.pdf]

Birch Pollen Related Pear Allergy: A Single Blind Oral Challenge Trial with 2 Pear Cultivars

Nicolette W. de Jong[1], Severina Terlouw[1], Frank E. van Boven[1], M.S. van Maaren[1], Marco W.J. Schreurs[2], Dianne B.P.M. van den Berg-Somhorst[3], Diederik Esser[3], Shanna Bastiaan-Net[3]

Supplementary material:

S1

Table 1 Indicative molecular weight of protein bands bound by IgE antibodies in patient sera; protein bands correspond to the western blots in Figure 3. Ce: 'Cepuna' pear; Co: 'Conference' pear; WB: western blot; the number of x's indicate the (by eye) estimated intensity of the coloration. Serum nr 9 was not available for western blotting.

	–15kDa		–19kDa		–22kDa		–27kDa		–35kDa		–41kDa		–45kDa	
WB#	Ce	Co	Ce	Co	Ce	Co	Ce	Co	Ce	Co	Ce	Co	Ce	Co
Negative control											(x)	(x)		
#1			x	x								(x)	x	x
#2													(x)	
#3			(x)	(x)									(x)	x
#4			xx	xx	xx	xx								xx
#5													xxx	xxx
#6		(x)											x	xx
#7													x	x
#8					x	x	x	xx					xxx	xxx
#10			x	x					xxx	x			xx	xx
#11			(x)	(x)									x	x
#12													xx	xxx
#13			x	x					x				xx	xx
#14													(x)	x
#15											x	x		

	–48kDa		–55kDa		–62kDa		–64kDa		–72kDa		–83kDa		–87kDa		–94kDa		–165kDa	
WB#	Ce	Co	Ce	Co	Ce	Co	Ce	Co	Ce	Co	Ce	Co	Ce	Co	Ce	Co	Ce	Co
Negative control																		
#1			(x)	(x)														
#2		x																
#3			(x)	(x)														
#4								x									x	
#5			x	x	x	x												
#6			x					x										
#7																		
#8			x	x	xx	xx			x	x								
#10			x								xx	xx						
#11																		
#12			x	x			x	x							x	x		
#13					x	x					(x)	(x)						
#14																		
#15			x		(x)	(x)			(x)	(x)			x	x				
